# Defining a Role for Webinars in Surgical Training Beyond the COVID-19 Pandemic in the United Kingdom: Trainee Consensus Qualitative Study

**DOI:** 10.2196/40106

**Published:** 2022-12-21

**Authors:** Emma Barlow, Wajiha Zahra, Jane Hornsby, Alex Wilkins, Benjamin M Davies, Joshua Burke

**Affiliations:** 1 Association of Surgeons in Training London United Kingdom; 2 Department of Trauma and Orthopaedics University Hospitals North Midlands Stoke-on-Trent United Kingdom; 3 Department of Neurosurgery University of Cambridge Cambridge United Kingdom

**Keywords:** webinars, surgical training, trainee consensus, teaching, training, integration, trainee experience, user experience, perception, education, medical education, surgical skills

## Abstract

**Background:**

The COVID-19 pandemic posed several challenges for surgical training, including the suspension of many in-person teaching sessions in lieu of webinars. As restrictions have eased, both prepandemic and postpandemic training methods should be used.

**Objective:**

This study investigates trainees’ experiences of webinars during the COVID-19 pandemic to develop recommendations for their effective integration into surgical training going forward.

**Methods:**

This project was led by the Association of Surgeons in Training and used an iterative process with mixed qualitative methods to consolidate arguments for and against webinars, and the drivers and barriers to their effective delivery, into recommendations. This involved 3 phases: (1) a web-based survey, (2) focus group interviews, and (3) a consensus session using a nominal group technique.

**Results:**

Trainees (N=281) from across specialties and grades confirmed that the COVID-19 pandemic led to an increase in webinars for surgical training. While there were concerns, particularly around the utility for practical training (80.9%), the majority agreed that webinars had a role in training following the COVID-19 pandemic (90.2%). The cited benefits included improved access or flexibility and potential standardization of training. The majority of limitations were technical. These perspectives were refined through focus group interviews (n=18) into 25 recommendations, 23 of which were ratified at a consensus meeting, which was held at the Association of Surgeons in Training 2021 conference.

**Conclusions:**

Webinars have a role in surgical training following the COVID-19 pandemic. The 23 recommendations encompass indications and technical considerations but also discuss important knowledge gaps. They should serve as an initial framework for ensuring that webinars add value and continue to evolve as a tool for training.

**Trial Registration:**

Chinese Clinical Trial Registry ChiCTR2200055325; http://www.chictr.org.cn/showprojen.aspx?proj=142802

## Introduction

The COVID-19 pandemic has significantly impacted many aspects of surgical training in the United Kingdom and the Republic of Ireland and has challenged the delivery of surgical training with the cancellation of examinations, training courses, and teaching programs, and a significant reduction in exposure to operative cases [1]. A study comparing surgical trainees’ operative logbook numbers in 2019 and 2020 reported an overall incident rate ratio (IRR) of 0.62, with exposure to elective surgery more affected (IRR 0.53) than emergency surgery (IRR 0.85) [2]. Subsequently, surgical training had to adapt, resulting in an accelerated shift in the use of digital learning environments by both trainees and trainers.

A webinar is defined as “a seminar conducted online” [3]. A seminar is a teaching method based on the Socratic dialogue of asking and answering questions with the word originating from the Latin *seminarium*, which means “seed plot” [4,5]. Webinars have evolved as a result of technological advancements leading to faster, more reliable internet connections and the use of video calls as a standard method of communication in today's society. As a result, digital learning was slowly being integrated into pedagogical learning methods to create new blended learning methods [6]. However, the COVID-19 pandemic presented a new opportunity for the use of webinars to provide remote learning for surgical trainees, and, as a result, they have become a popular and increasingly prevalent training tool [4]. One review of the use of webinars for training in plastic surgery indicated an increase of 12,017% in the number of webinars relevant to this specialty post lockdown in the United Kingdom on the March 23, 2020 [4].

While the pandemic forced training to transition to the internet, the question remains if and how webinars should be integrated within surgical training going forward as the National Health Service’s services start to recover and surgical training adapts to the new norm. The Joint Committee on Surgical Training quality indicators for Higher Surgical Training states that trainees should have a minimum of 2 hours of facilitated formal teaching every week [7].

This study, led by the Association of Surgeons in Training (ASiT), aimed to identify the role of webinars in surgical training, both during and after the COVID-19 era, and consider what, in a best-case scenario, their potential integration into surgical training would look like in the future. To achieve this, the following objectives were set: (1) to investigate the strengths and weaknesses of webinar-based training for surgical specialties, (2) to establish the limitations of webinars and the barriers faced when integrating them into surgical training, and (3) to propose recommendations for its integration within surgical training.

## Methods

### Overview

This study was led by the ASiT via a steering committee of surgical trainees. The ASiT is an independent professional organization (registered charity number 1196477), with a membership of over 3500 surgical trainees, that promotes excellence in surgical training. The organization represents and supports trainees across all surgical specialties and training grades on a regional and national level in the United Kingdom and the Republic of Ireland and is the largest representative body for surgical trainees. The ASiT was originally founded in 1976 and is independent of, but works with, the National Health Service, General Medical Council, Surgical Royal Colleges, the Joint Committee on Surgical Training, and the Trainee Surgical Specialty Associations.

This study used mixed qualitative methods in accordance with COREQ (consolidated criteria for reporting qualitative research) guidelines [8] to consolidate the arguments for and against webinars and the drivers and barriers to their delivery in surgical training. This involved 3 phases: (1) a web-based survey, (2) focus group interviews, and (3) a consensus session at the annual ASiT 2021 international conference.

### Phase 1: Web-Based Survey

A survey was developed to gather wider trainee perspectives on webinars in surgical training. It was built using Qualtrics (Qualtrics XM) in accordance with the published CHERRIES (Checklist for Reporting Results of Internet E-Surveys) guidelines on conducting web-based surveys with the aim of establishing broad opinion and key themes concerning the use of webinars before and during the COVID-19 pandemic [9-11]. The survey contained binomial, multiple-choice, Likert-scale questions and those with free-text responses (Multimedia Appendix 1) and was developed on the basis of consensus from the steering group. The survey was peer reviewed and piloted by the ASiT council prior to dissemination, where 1 question was omitted and 2 questions reworded, resulting in a final set of 26 questions. The survey was sent out to all ASiT members via the ASiT mailing list (MailChimp) and advertised via ASiT social media channels (Facebook and Twitter). Survey completion was voluntary and open to current and future surgical trainees of all grades and specialties. All responses were collected with informed consent provided by responders at the time of completion, and anonymized over a 6-week period (December 3, 2020, to January 14, 2021) without sampling. Thereafter, the survey was removed from the ASiT website. Descriptive statistics were used to aggregate data from predefined questions. Responses to a final free-text question—“Please feel free to write any other comments regarding your experience of teaching/training via webinars.”—were subjected to inductive thematic analysis by 2 authors (AW and JH) independently.

### Phase 2: Focus Group Interviews

Trainees interested in participating in the interview stage were contacted via email with further study information and available interview slots between Monday, February 8, and Sunday, February 14, 2021. The interviews were arranged as 30-minute focus group sessions with up to 4 participants in each group and one member of the study group present to facilitate the session, prompt discussions, and answer queries. These sessions were conducted as web-based video calls using Zoom (Zoom Video Communications). Each session was recorded and transcribed with participants consent. Interviews were conducted using structured discussion. Interview transcripts and recordings were analyzed using thematic analysis, which produced 4 themes including strengths, limitations, drivers, and barriers, with focused questions generated through steering group consensus from the initial survey responses. All focus group participants were offered collaborative authorship [12].

### Phase 3A: Formation of Consensus Statements

This project used a transparent consensus process [13]. Based on the findings of the survey and focus group interviews, a preliminary list of consensus statements (Multimedia Appendix 2) was formed by the steering committee (EB, WZ, JH, AW, BD, and JB), and refined with input from the ASiT council, into a final list for presentation during the consensus meeting (Multimedia Appendix 3). These were designed to capture the perspectives gathered so far and structured around the following core areas: the role, timing, conduct, content, opportunities, and knowledge gaps for webinar use.

### Phase 3B: Consensus Meeting

The consensus meeting was held at the 2021 ASiT annual international conference, which was the first ASiT conference to be held remotely. It was advertised as free for any conference delegate via the conference platform (MedAll) and program, the ASiT website [14], and social media platforms. A Google Form (Google LLC) was shared among attendees and used to record the name, gender, specialty, grade, and deanery of all participants who attended the meeting. The session was held using Zoom, and discussion during the meeting took place both verbally and using the question-and-answer function. Slido (sli.do s. r. o.) was used during the consensus session to present each consensus statement and permit attendees to vote. Votes were binary (yes/no or agree/disagree). For a statement to be ratified, 70% or more of the consensus participants had to vote in agreement (yes/agree). The cutoff of 70% was based on the Grading of Recommendations Assessment, Development and Evaluation approach and agreed on by the study group prior to the session [15,16]. The Slido software automatically stored the results and facilitated their export for data analysis.

### Ethics Approval

According to the Health Research Authority Guidance, ethics approval was not sought for this study.

## Results

### Phase 1: Web-Based Survey

#### Demographics

The survey was sent to 2790 ASiT members. The total number of survey responses was 281, with a response rate of 9.9%. In total, 278 complete responses were included in the final analysis, as incomplete responses were excluded.

All training regions in the United Kingdom and the Republic of Ireland were represented. In total, 56.7% of respondents were female. The majority of respondents were trainees in general surgery (43.2%), followed by orthopedics (21.6%), with responses from trainees in all other surgical specialties. There were responses from core surgical trainees (41.0%), specialist registrars (35.6%), foundation trainees and medical students (17.9%), specialty doctors or associate specialists (3.8%), and post–certificate of completion of training fellows 1.7%; Multimedia Appendix 4).

#### Key Findings

**Figure 1:**
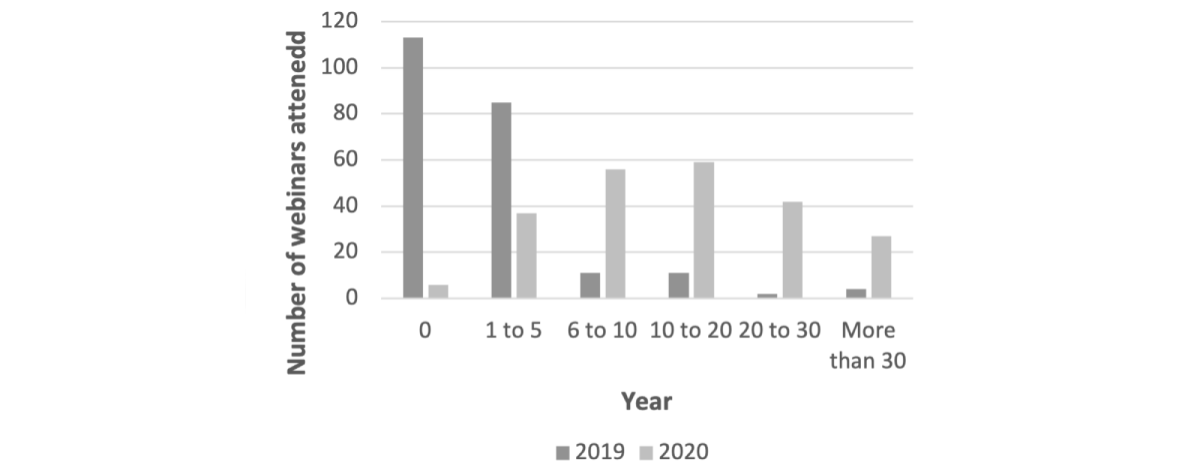
Graphical representation of the number of webinars attended by survey respondents in 2019 and 2020.

Respondents reported attending more training webinars in 2020 than in 2019 (Figure 1) and 96.5% of respondents agreed that this change was due to the COVID-19 pandemic. Overall, 98.9% of respondents agreed that webinars have become a standardized format for surgical teaching to replace face-to-face teaching during the COVID-19 pandemic. However, 80.9% agreed that webinars cannot fulfil the practical aspects of surgical training.

Specific reasons for attending more webinars included webinars being the only resource available (28.9%), more webinars being available (23.4%), to meet specific training requirements (15.8%), more awareness of webinars (15.6%), and webinars being an effective way to use time (14.3%). Only 10 respondents did not attend webinars, and the reasons for this included that there are too many webinars, so it is difficult to choose which ones to attend, and that they seem less effective for surgical training. The main factors that are considered when deciding to attend a webinar include the topic (27.6%), training requirements (20.6%), the speaker (15.4%), and cost (15.6%).

Regarding the delivery of webinars (Multimedia Appendix 5), the preferred duration of a webinar for 69.1% of respondents was 30 to 60 minutes. The most popular time of webinars was weekdays out of working hours. The majority of respondents had 1-2 hours available to attend webinars each week.

Respondents were asked to rate aspects about the effectiveness of webinars on Likert scales. Overall, the mean score for the webinar as a format for surgical training during the COVID-19 pandemic was 6.8 (SD 1.8) out of 10. The organization and structure of webinars had a mean score of 7 (SD 1.86) out of 10. The interaction between speakers and participants had a mean score of 5.2 (SD 2.22) out of 10, and technical aspects scored 6.4 (SD 2.29) out of 10. Meeting the demand of practical education scored poorly at 4.3 (SD 2.75) out of 10. Most people (44.5%) felt that the topic of the webinar determined whether it would be as effective as face-to-face teaching. Overall, 91.2% of respondents felt that they were more likely to ask questions during face-to-face teaching than during a webinar and that they were more likely to pay for face-to-face teaching.

Going forward, 90.2% of respondents were very likely, likely, or somewhat likely to attend training webinar after the COVID-19 pandemic. Respondents thought that webinars and web-based learning would be most useful for exam preparation, journal clubs, annual reviews of competency progression), and supervisor meetings (Multimedia Appendix 5).

In total, 106 respondents provided free-text answers when asked if they had any other comments about webinars. The themes from these responses were identified as demonstrated in Table 1. 

**Table 1:** Themes identified from the free-text answers in the initial web-based survey.

Theme	Example quotes
Webinars improve accessibility to teaching	“I would watch a webinar of an interesting topic given by an institution very far from where I am based. In fact, I may be even more likely to do than I would to attend physically as the costs would be much lower.” “World experts can be called upon to give a webinar on a given topic.” “A great equaliser for those with kids etc to be able to attend without travelling.”
Webinars are useful for examination preparation	“I think the training webinars focusing on required curriculum (exam/FRCS style preparation) and related topics have been very useful and are as good as in person teaching.”
Webinars are less engaging	“I am more prone to distraction and lack of engagement in webinars than in face-to-face teaching.”
Webinars cannot replace teaching for practical skills	“A lot of practical/surgical stuff involving hands on technique cannot be taught via webinars and therefore webinars are in no way a substitute for face-to-face training but merely an adjunct.”
Trainees have to use their spare time and own space for webinars	“Too many mandatory/highly useful webinars/sessions held outside of working hours.” “There is no down time now and people are expected to be available for teaching/meetings whether at work, or off work with illness/zero day/annual leave.”

### Phase 2: Focus Group Interviews

#### Demographics

In total, 18 trainees (outwith the steering committee facilitators) participated in 8 semistructured interviews conducted in February 2021. The interviewees were trainees in general surgery (n=7), trauma and orthopedics (n=5), otolaryngology (n=1), plastic surgery (n=1), urology (n=1), and vascular surgery (n=1). The foundation trainees (n=2) did not declare their intended surgical specialty. The training grades of the interviewees were specialist registrars (n=8), core surgical trainees (n=6), foundation trainees (n=3), and senior house officer–level staff grade (n=1; see Multimedia Appendix 4).

#### Key Findings

Interview responses generally supported survey findings with regards to strengths, limitations, drivers, and barriers. Recurrent themes of accessibility, timing, and technical aspects were explored in more detail.

#### Accessibility and Cost

Avoiding travelling to remote locations was reported as beneficial for time, financial, and environmental reasons. Accessibility to worldwide experts offered an opportunity to access international speakers and panels usually only available at major conferences, while avoiding the associated cost and study leave. On a regional level, webinars allowed deanery teaching programs to be combined to maintain high-quality speakers despite reduced speaker availability. There was considerable heterogeneity in individual experience, a minority of interviewees reported formal protected time and spaces for locally arranged webinars, this may not be practical for national or international events.

#### Timing of Webinars

Interviewee preferences for timing of webinars varied with their individual circumstances. Some identified midweek evenings as a time with less conflicting demands. Concerns were raised that a reliance on out-of-hours webinars disenfranchised those with family or other commitments and could contribute to burnout. One trainee highlighted the diversity of views, explaining that as she lived away from her family during the working week, she preferred to watch webinars during weekday evenings to keep her weekends and working days free. Some expressed a preference for watching webinars at home, others found it difficult to focus with multiple distractions. Access to study leave for webinars during working hours was reported as limited.

#### Recordings

The ability to view recordings later was welcomed as adding flexibility, with the reservation that recordings lose interactivity, particularly the opportunity to ask questions. Repeat recordings may not remain up to date with current evidence and guidelines and may need to be reviewed. Archiving of content is variable and could be improved.

#### Technical Aspects

Many interviewees described variation in technical fluency between presenters. Live polls, chat, and question-and-answer functions were highlighted as improving interactivity. Equipment and bandwidth problems were common; in one example, an anatomy session from a dissecting room had insufficient resolution to identify structures. This may represent the learning curve of adopting new teaching methods and could be addressed through targeted training on the use of web-based platforms, and appropriate technical support. When available, a session chair can have a valuable role to set expectations and manage features (including muting and unmuting, screen sharing, and reviewing audience chat) to allow the presenter to focus on presenting.

#### Future Opportunities

To counter the loss of social interaction, a suggestion was made that a hybrid approach with remote access to face-to-face teaching offers a solution for deanery teaching to reduce the need to travel. Small groups meeting locally to access remote teaching would allow some element of networking and social interaction.

### Phase 3: Consensus Session

#### Demographics

The consensus session was attended by 33 delegates, of whom 32 completed the demographics questionnaire. In total, 20 (62.5%) participants were female and 12 (37.5%) were male. A range of training grades and surgical specialties were represented among the consensus session cohort with specialist registrars (ST3-8; 43.8%) the most represented grade and general surgery (37.5%) the most represented specialty (see Multimedia Appendix 4).

#### Voting Results

Of the 25 Statements, 23 obtained at least 70% approval from the consensus meeting participants (Table 2) and are included in the recommendations of this paper.

**Table 2:** List of consensus statements and the percentage of consensus votes received.

Consensus statement	Consensus rate, %
Webinars have a role in surgical training but should not completely replace face-to-face training	100
Webinars are considered a good option for theoretical knowledge	94
Webinars are considered a good option for examination preparation (mock interviews or viva voces)	88
Webinars are considered a good option for training administration (eg, work-based assessments, journal clubs, and annual reviews of competency progression)	82
Webinars are currently considered a poor option for practical skills	88
Webinars are currently considered a poor option for simulation training (eg, advanced trauma life support)	88
Webinars are currently considered a poor option for communication skills	42
The following is considered best practice for delivering surgical training webinars: they should be delivered live (not prerecorded)	70
The following is considered best practice for delivering surgical training webinars: they should be recorded (available for playback)	94
The following is considered best practice for delivering surgical training webinars: they should be supported by an information technology specialist for troubleshooting and support	97
The following is considered best practice for delivering surgical training webinars: they should incorporate interactive elements, including a chat box, polls, or breakout room	97
The following is considered best practice for delivering surgical training webinars: they should be effectively archived and easily retrievable for future review	94
The following is considered best practice for delivering surgical training webinars: a certificate of attendance should be issued to allow trainees to log professional development	100
The following is considered best practice for the timing of surgical training webinars: they should not exceed one hour	73
The following is considered best practice for the timing of surgical training webinars: they should be eligible for study leave and be delivered within protected teaching time	100
Webinars delivered during evenings may increase trainee engagement by avoiding clashing with clinical commitments; however, this may disadvantage trainees who have families, long commutes, or other extracurricular commitments	97
Payment for webinars, outside of core content delivered as part of their training program, is acceptable to surgeons in training, but it should reflect the fair costs of hosting the webinar	48
Surgical trainers should be provided with resources and training to develop their digital teaching skills	100
Webinars offer opportunities to improve access and equality of training for trainees (eg, through delivery regionally, nationally, or internationally), and this should be explored further	97
The mechanism by which webinar attendance is recognized or accredited should be clarified	97
The value of structured webinars (ie, a series of webinars) aligned with surgical curricula is uncertain but should be explored further	97
While access to learning resources can be improved with webinars, the lost opportunities for networking, team building, or socializing and their implications are uncertain and should be explored further	100
Increased participation in web-based training out of normal working hours and how this contributes to trainee burnout should be explored further	100
Adjuncts to support web-based or remote practical skills training are evolving; therefore, the role or value of webinars for practical skills training should be revisited	97
A hybrid approach, using both face-to-face and web-based methods, may be the future of surgical training and should be explored further	100

## Discussion

### Principal Findings

This initiative has produced the first trainee-led, consensus-based recommendations on the use of webinars in surgical training with unanimous agreement that webinars have a role in future surgical training. Both practical and communication training was considered poorly suited, although it was acknowledged that this may change as methods of remote training evolve. These 23 consensus recommendations should support their continued and effective implementation and iteration.

A role for webinars in surgical training was anticipated, in line with the positive experiences that have been increasingly published across health care disciplines globally [17]. Blythe and Thompson [18] report the successful implementation of a novel web-based surgical teaching program in Northern Ireland with support from the Royal College of Surgeons Edinburgh. The program was designed to meet the needs of core surgical trainees during the COVID-19 pandemic owing to cancellation to face-to-face teaching and used both videoconferencing software and web-based webinars. As a result of the positive feedback received, the course has been further developed into the primary surgical teaching method for the core surgical training cohort of 2020-2021 in Northern Ireland. This shows the sustainability and value of remote education going forward [18].

Webinars allow the remote delivery of expert teaching to a large number of trainees simultaneously, and recording these sessions offers further opportunity to increase the audience to people unable to attend live. This also provides trainees the option of revisiting the webinar to consolidate their learning or revise it at a later date. As observed during the development of our ASiT recommendations, accessibility and convenience are the commonly cited reasons for their use. The Virtual ACCESS conference (a core-trainee–led web-based conference to enhance surgical succession) surveyed delegates and found that the major factors that attracted attendees to their web-based conference were that the conference was free (91/130, 70%), allowed an opportunity to present (81/130, 62.3%), and did not require travel (78/130, 60%) [19].

### Limitations

However, as highlighted in our process, webinars elsewhere also have their limitations. This seems to be largely around variation in technical delivery [20]. This was a large focus of the feedback received during our initiative and subsequently recognized within the recommendations; for example, around timing, duration, technical support, and archiving. An expectation of (unpaid) availability out of hours has implications for the costs of surgical training [21]. Although not specifically for webinars, technical challenges are a commonly cited barrier in telemedicine and are more frequently studied [20]. The preference for live delivery was also noteworthy, given that many web-based education initiatives use recorded videos [22,23]. As the role of guidelines is to improve experience by standardizing practice with the best available evidence [24], it is hoped that these recommendations can therefore have an immediate and positive impact.

However, there are some important limitations to acknowledge in our process. First, this process only captured the perspective of trainees. Therefore, it did not incorporate evidence from medical education, such as the comparative effectiveness of webinars, nor did it incorporate the perspective of surgical trainers specifically [25]. We also acknowledge a low response rate. The collaborative coauthorship model used and the outlined study design were chosen to incentivize participation. While these must be considered, it is worth recognizing that response rates parallel those of similar surveys [26-28] and perspectives across disciplines and training stages were still represented. Further, trainees are typically involved in education themselves and will have a trainer’s perspective.

Second, while most recommendations reached near unanimous consensus, there were inconsistencies. For example, while the initial survey suggested that trainees did not feel that communication skills were well suited to webinars, this did not become a consensus recommendation. This discordance may reflect an evolving perspective on webinars and communication as the initial concerns centered around being unable to recognize nonverbal communication in a web-based environment. Given that clinical practice has also transitioned to many web-based consultations [29], it is likely that remote teaching will have a more relatable role. Similarly, while practical training was felt to be better suited to alternative teaching methods, this was not unanimous, with agreement that, particularly as training tools develop [30], the role of webinars in practical training be investigated further. Of note, Fehervari et al [31] reported the first web-based, large-scale, surgical skills course that compared different web-based teaching methods and was validated against the gold standard of face-to-face teaching. The published feedback showed the success of this web-based course as delegates felt that the event met the required standards of a high-quality surgical teaching course and did not feel that face-to-face teaching would have been more appropriate despite the course focusing on practical skills [31]. This differs from the results of our consensus session and shows that there is scope for further research into the utility of webinars and web-based teaching for practical training.

The inconsistencies likely reflect the rapidly changing landscape of webinars in surgical education [20], and the need to iterate these recommendations as evidence and experience changes [29]. Based on the uncertainties identified during this process, a number of key knowledge gaps for clarification were identified, which too should help to guide their optimization.

Therefore, while we consider these recommendations to have been formed through an inclusive and structured process, and of immediate value, as with any form of recommendation, they should be considered a starting point that will require update over time.

### Conclusions

This is the first initiative to produce consensus-based recommendations on the role and use of webinars within surgical training. These recommendations were produced by trainees and should serve as an initial framework for ensuring that they add value to surgical training in the future.

## Appendix

Multimedia Appendix 1 Initial online survey created using Qualtrics.

Multimedia Appendix 2 List of preliminary anticipated statements prior to review and finalisation.

Multimedia Appendix 3 List of finalised statements presented during the consensus meeting.

Multimedia Appendix 4 Table of participant demographics for each stage of the study.

Multimedia Appendix 5 The Delivery of Webinars a) Graph showing the number of hours trainees have available to attend webinars each week b) Graph showing trainees’ preferred timings of webinars c) Graph showing trainees’ preferred duration of webinars d) Graph showing the preferred setting for webinars/virtual learning to be used in the post pandemic surgical training environment.

## Abbreviations

ASiT: Association of Surgeons in Training
CHERRIES: Checklist for Reporting Results of Internet E-Surveys
COREQ: consolidated criteria for reporting qualitative research
