# Supplementary material for: Defining a Role for Webinars in Surgical Training Beyond the COVID-19 Pandemic in the United Kingdom: Trainee Consensus Qualitative Study
Source: JMIR Med Educ. 2022 Dec 21;8(4):e40106. doi: 10.2196/40106 (PMC9813811; doi:10.2196/40106)
Supplement: Multimedia Appendix 1 [file mededu_v8i4e40106_app1.docx]

# **Supplementary Data A**

*Initial online survey created using Qualtrics.*

**Beyond COVID-19: Defining a role for Webinars in Surgical Training**

Q1. This survey forms part of a national project assessing the perceptions of surgical trainees on webinar teaching during the COVID-19 pandemic. This will help inform whether we can proceed with this mode of teaching post-pandemic. If you are currently a surgical trainee or a junior doctor on surgical rotation, you are eligible to take part. You can still take part even if you have not attended any webinars.

Taking part is completely voluntary and confidential. Any responses you provide will be anonymized. Taking part involves completing a short survey. The study is being run by Association of Surgeons in Training (ASIT). The first question of the survey will ask you to confirm your consent to participate in the study. Trainees who take part in the focus group/interview stage of this project will be named as collaborators on any resulting papers.

Please contact us with any questions you may have: wajiha.zahra@nhs.net

Thank you very much for your time and support.

Q2 Do you consent to participate in this study?

o Yes

o No

*Skip To: End of Survey If Do you consent to participate in this study? != Yes*

Q3 In which geographical region are you currently working?

o East of England

o East Midlands

o Kent, Surrey and Sussex

o London

o North East

o Northern Ireland

o North West

o Peninsula

o Republic of Ireland

o Severn

o Scotland

o Thames Valley

o Wales

o West Midlands

o Wessex

o Yorkshire and Humber

Q4 In which speciality do you currently work?

o General Surgery

o Urology

o Trauma and Orthopaedics

o Neurosurgery

o Cardiothoracic Surgery

o Plastic Surgery

o Ear, Nose and Throat

o Vascular Surgery

o Paediatric Surgery

o Obstetrics and Gynaecology

o Ophthalmic Surgery

o Oral and Maxillofacial Surgery

o Other

Q5 What is your training grade?

o Foundation Trainee (FY1 or FY2)

o Core Surgical Trainee (CT1-CT2)

o Specialist Registrar (ST3-ST8)

o Post- CCT Fellow

o Specialty Doctor/Associate Specialist

Q6 What is your sex?

o Male

o Female

o Prefer not to say

Q7 Approximately how many training webinars did you attend in 2019 including mandatory teaching?

o 0

o 1 to 5

o 6 to 10

o 10 to 20

o 20 to 30

o More than 30

Q8 Approximately how many training webinars have you attended in 2020 including mandatory teaching?

o 0

o 1 to 5

o 6 to 10

o 10 to 20

o 20 to 30

o More than 30

Q9 Have you attended more training webinars as a result of the COVID-19 pandemic?

o Yes

o No

*Skip To: Q11 If Have you attended more training webinars as a result of the COVID-19 pandemic? = No*

*Display This Question:*

*If Have you attended more training webinars as a result of the COVID-19 pandemic? = No*

Q11 I do not attend webinars, because?

▢ I do not have time

▢ They seem less effective for surgical training

▢ There are too many webinars going on - difficult to chose

▢ I am done with my training- I don't need to attend

▢ Other

*Skip To: End of Survey If Condition: I do not attend webinars, b... Is Equal to 1. Skip To: End of Survey.*

Q10 If yes, what made you attend more training webinars during COVID-19 pandemic?

▢ Only teaching source available

▢ Requirement for training

▢ Effective way to utilize time

▢ Socializing

▢ More webinars available

▢ More awareness of webinars

▢ Other

Q12 How many hours of training webinars do you have time to attend per week?

o 0

o 1 to 2

o 2 to 4

o 5 or more

Q13 What is your preferred duration of webinar?

o 30 minutes to 1 hour

o 1 to 2 hours

o More than 2 hours

o Time doesn't matter

o 30 minutes or less

Q14 On which days do you prefer Webinars?

o Weekdays during working hours

o Weekdays out of working hours

o Weekends

o No preference

o Watch the recordings

Q15 What factors do you consider when deciding to attend a webinar?

▢ Topic

▢ Speaker

▢ Organisation

▢ Recommendation

▢ Training need

▢ Cost

▢ Other

Q16 Overall, how would you rate the webinar as a format for surgical training during the COVID-19 pandemic?


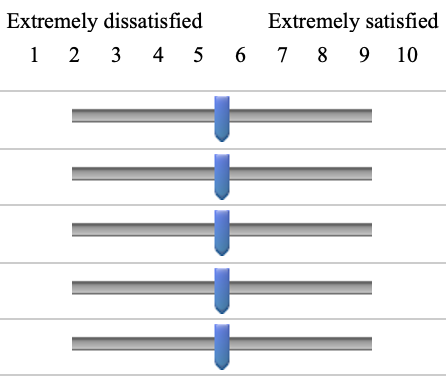


Q17 How would you rate the following aspects of training webinars?


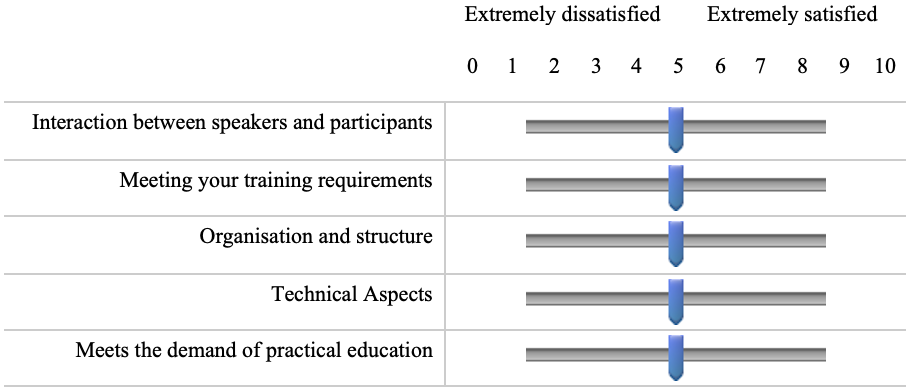


Q18 Do you find training webinars as effective as the face to face teaching?

o Prefer webinars

o Prefer face to face teaching

o No preference

o Depends on the topic

Q19 Would you prefer attending a webinar or a face to face teaching session post-COVID?

o Prefer webinars

o Prefer face to face teaching

o No preference

o Depends on the topic

Q20 Are you more or less likely to ask questions during a webinar or face to face teaching?

o More likely during webinar

o More likely during face to face teaching

o Equally likely - I would ask questions regardless of the format

o Equally unlikely - I do not usually ask questions

Q21 Are you more or less likely to pay for a training webinar or for face to face teaching?

o More like for webinar

o More likely for face to face teaching

o Equally likely if it is a training requirement

o Equally unlikely

Q22 'Webinars cannot fulfill the practical aspect of surgical training'. Do you agree with this statement?

o Agree

o Neither agree nor disagree

o Disagree

Q23 "During the COVID-19 pandemic, webinars have become a more common format of my surgical teaching than face to face teaching". Do you agree with this statement?

o Yes

o No

o Teaching sessions are still same

Q24 How likely are you to attend training webinars post-COVID?

o Very likely

o Likely

o Somewhat likely

o Undecided

o Somewhat unlikely

o Unlikely

o Very unlikely

Q25 In what settings, if any, do you think webinars/virtual learning should be used in a post-pandemic training environment?

▢ Exam Preparation

▢ Virtual Examinations

▢ Surgical Skills Training

▢ Journal Clubs

▢ Work Based Assessments

▢ ARCP

▢ Supervisor Meetings

▢ Other

Q27 Please feel free to write any other comments regarding your experience of teaching/training via webinars.

Q28 If you would be happy to take part in a telephone interview or focus group in the future to explore this topic further, please provide your email address. Many thanks for participating in this survey.

________________________________________________________________
