# Supplementary material for: Defining a Role for Webinars in Surgical Training Beyond the COVID-19 Pandemic in the United Kingdom: Trainee Consensus Qualitative Study
Source: JMIR Med Educ. 2022 Dec 21;8(4):e40106. doi: 10.2196/40106 (PMC9813811; doi:10.2196/40106)
Supplement: Multimedia Appendix 2 [file mededu_v8i4e40106_app2.docx]

# **Supplementary Data B**

*List of preliminary anticipated statements prior to review and finalisation.*

| **Theme** | **Statement** |
| --- | --- |
| **Role and Conduct** | Webinars have a role in surgical training, but should not completely replace face-to-face training  Webinars are considered good options for:   - Theoretical Knowledge - Training Administration (E.g. Work Based Assessments and Annual Review of Competence Progression) - Exam Preparation (Mock Interviews or VIVA)   Webinars are currently considered poor options for:   - Practical Skills - Simulation Training (E.g. ATLS) - Communication Skills   The following is considered best practice for delivering surgical training webinars, with regard:   - be delivered live (not pre-recorded) - be recorded (available for playback) - be supported by an information technology specialist, for troubleshooting and support - incorporate interactive elements, including chat box, polls or breakout rooms - be effectively archived, and easily retrievable for future review - a certificate of attendance should be issued to allow trainees to log CPD   The following is considered best practice for the timing of surgical training webinars, with regard:   - should not exceed 1 hour - should be eligible for study leave and/or delivered within protected teaching time - webinars delivered during evenings reduce inequality by making it easier to attend live webinars around clinical commitments. - webinars delivered during evenings may increase inequality in access to teaching by disadvantaging trainees with families, long commutes or both.   Payment for webinars, outside of core content delivered as part of their training programme, is acceptable to surgeons in training, but should reflect the fair costs of hosting the webinar.  Surgical trainers should be provided with resources and/or training to develop their virtual teaching skills. |
| **Recommended Knowledge Gaps for Clarification** | Webinars offer opportunities to improve access and equality of training for trainees (e.g. through delivery regionally, nationally and/or internationally), and this should be explored further.  The mechanism by which webinar attendance is recognised or accredited, should be clarified.  The value of structured webinars (i.e. a series of webinars) aligned with surgical curricula is uncertain, but should be explored further.  Whilst access to learning resources can be improved with webinars, the lost opportunities to network, team build or socialise, and their implications are uncertain, and should be explored further.  Adjuvants to support virtual and/or remote practical skills training are evolving, and therefore the role / value of webinars for practical skills training should be revisited. |

# 
