# Supplementary figures and images for: Defining a Role for Webinars in Surgical Training Beyond the COVID-19 Pandemic in the United Kingdom: Trainee Consensus Qualitative Study
Source: JMIR Med Educ. 2022 Dec 21;8(4):e40106. doi: 10.2196/40106 (PMC9813811; doi:10.2196/40106)

# **Supplementary Data D**

*Table of participant demographics for each stage of the study.*


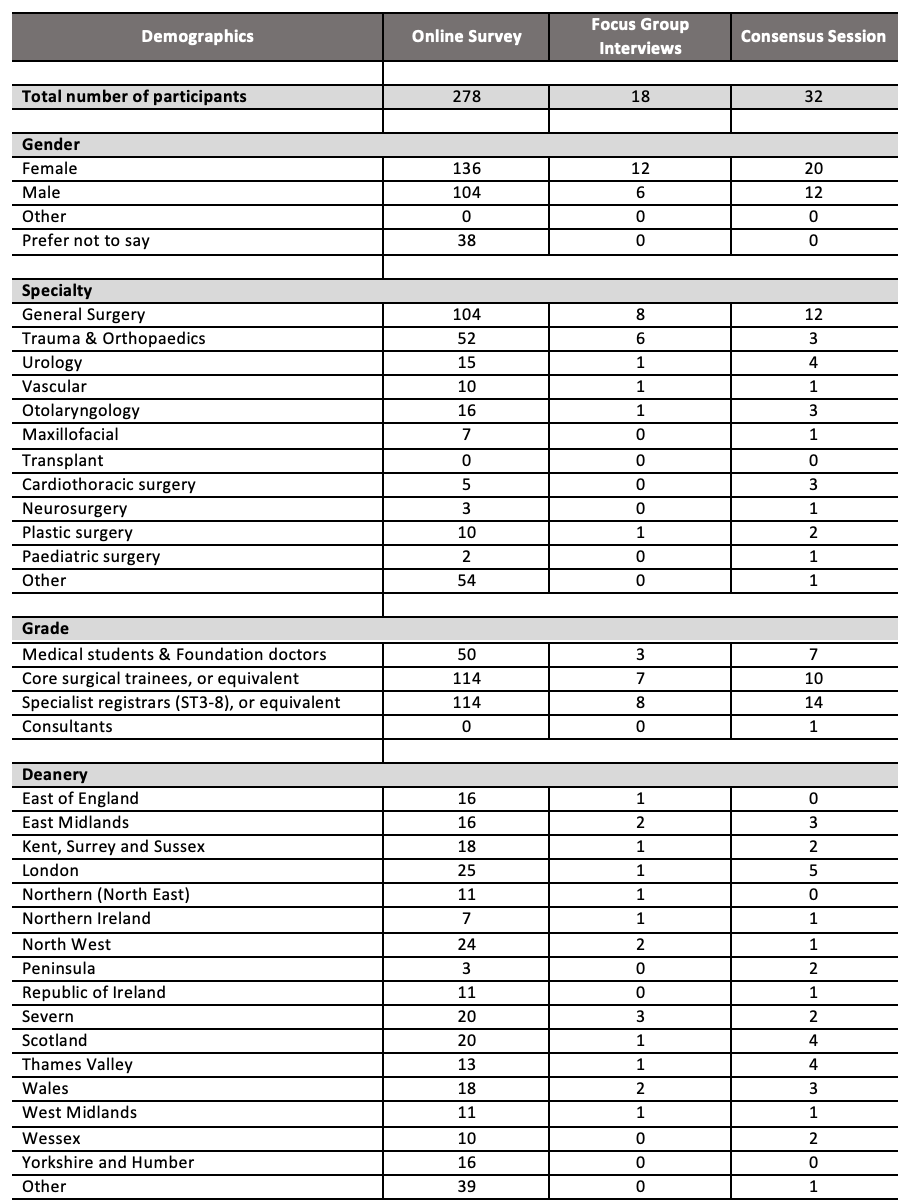

Supplement: Multimedia Appendix 4 [file mededu_v8i4e40106_app4.docx]
