# Supplementary material for: Defining a Role for Webinars in Surgical Training Beyond the COVID-19 Pandemic in the United Kingdom: Trainee Consensus Qualitative Study
Source: JMIR Med Educ. 2022 Dec 21;8(4):e40106. doi: 10.2196/40106 (PMC9813811; doi:10.2196/40106)
Supplement: Multimedia Appendix 5 [file mededu_v8i4e40106_app5.docx]

# **Supplementary Data E**

*The Delivery of Webinars*

*a) Graph showing the number of hours trainees have available to attend webinars each week.*

*b) Graph showing trainees’ preferred timings of webinars.*

*c) Graph showing trainees’ preferred duration of webinars.*

*d) Graph showing the preferred setting for webinars/virtual learning to be used in the post pandemic surgical training environment.*


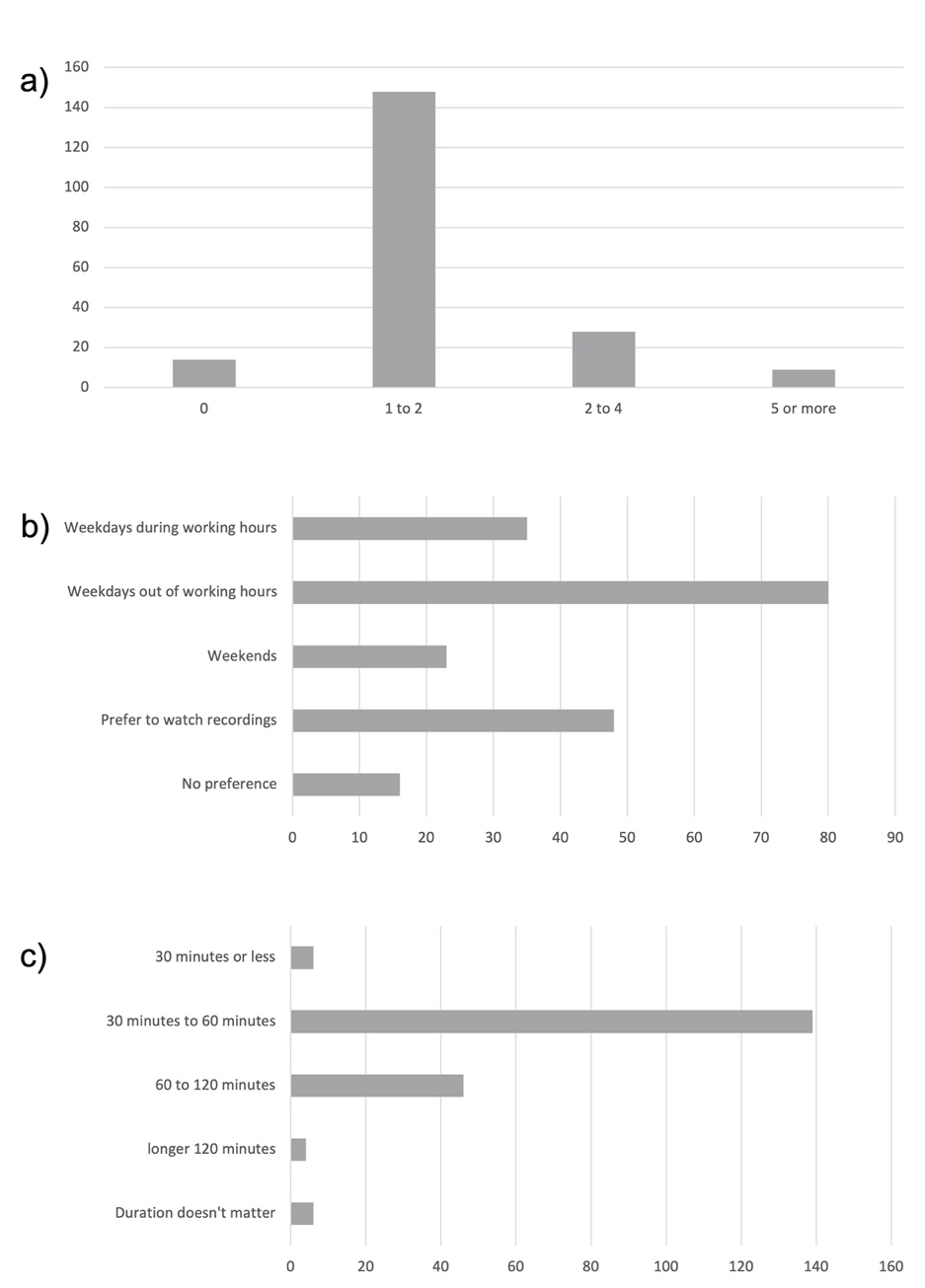


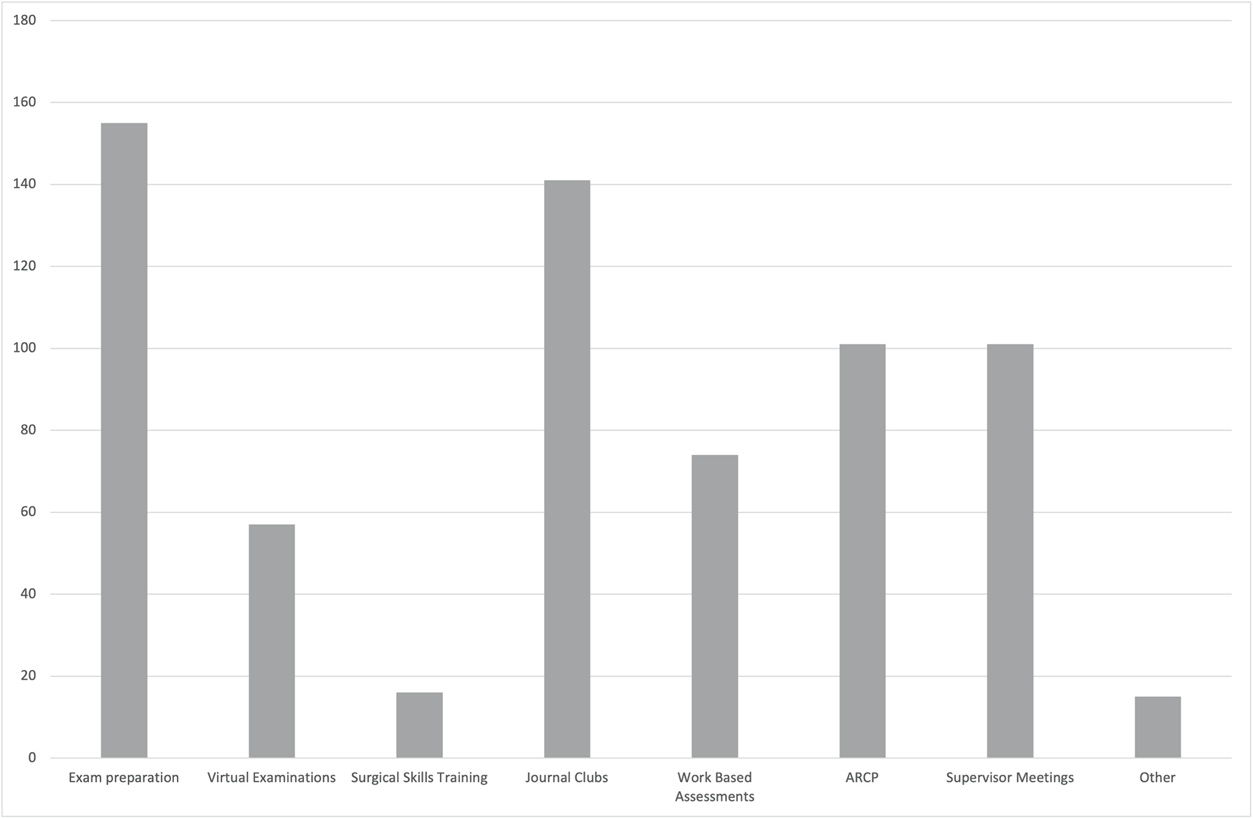


d)
